# Delay discounting correlates with depression but does not predict relapse after antidepressant discontinuation

**DOI:** 10.1038/s41380-025-03402-5

**Published:** 2026-01-08

**Authors:** Doron Elad, Giles W. Story, Isabel M. Berwian, Klaas E. Stephan, Henrik Walter, Quentin J. M. Huys

**Affiliations:** 1 Ruth and Bruce Rappaport Faculty of Medicine, Technion-Israel Institute of Technology, Haifa, Israel; 2 Division of Psychiatry, University College London, London, UK; 3 Max Planck-UCL Centre for Computational Psychiatry and Ageing Research, London, UK; 4 Lancashire and South Cumbria NHS Foundation Trust, Preston, UK; 5 Research Department of Clinical, Educational & Health Psychology; University College London, London, UK; 6 Translational Neuromodeling Unit (TNU), Institute for Biomedical Engineering, University of Zurich and ETH Zurich, Zurich, Switzerland; 7 Max Planck Institute for Metabolism Research, Cologne, Germany; 8 Charité – Universitätsmedizin Berlin, corporate member of Freie Universität Berlin and Humboldt-Universität zu Berlin, Department of Psychiatry and Psychotherapy, Berlin, Germany; 9 Applied Computational Psychiatry Lab, Mental Health Neuroscience Department, Division of Psychiatry and Max Planck UCL Centre for Computational Psychiatry and Ageing Research, Queen Square Institute of Neurology, UCL, London, UK

**Keywords:** Neuroscience, Psychology

## Abstract

Approximately one third of people with Major Depressive Disorder (MDD) experience a relapse within six months of discontinuing antidepressant medication (ADM), however, reliable predictors of relapse following ADM discontinuation are currently lacking. A putative behavioural predictor is delay discounting, which measures a person’s impatience to receive reward. Previous studies have linked delay discounting to both MDD and reduced serotonergic function, rendering it a plausible candidate predictor. In this multi-site study we measured delay discounting in participants with remitted MDD (*N* = 97), before and within six months after discontinuation of ADM, and in matched controls without a lifetime history of MDD (*N* = 54). Using predictive models, we tested whether either baseline discounting, or an early change in discounting following ADM discontinuation, predicted depressive relapse over a six month follow up period. We also tested differences between remitted MDD and control groups in delay discounting at baseline, and associations between discounting and depressive symptoms. We found that the remitted MDD group, compared to the control group, showed significantly higher (p < 0.05; Cohen’s *d* = 0.34) discounting at baseline. In addition, baseline discounting was positively correlated with depression rating scores (Spearman ρ = 0.24). However, delay discounting did not increase following ADM discontinuation. Neither baseline discounting, nor a change in discounting following ADM discontinuation, predicted subsequent depressive relapse. We conclude that delay discounting is elevated in remitted MDD treated with antidepressant medication. However, delay discounting neither increases following ADM discontinuation, nor does it prospectively predict depressive relapse. These results suggest that delay discounting in Major Depressive Disorder has little relationship with illness trajectory following ADM discontinuation.

## Introduction

Depressive disorders are estimated to be among the largest contributors to years lived with disability worldwide [1, 2]. This huge burden of morbidity is largely attributable to the chronic or recurring pattern [3, 4] that often characterizes depression. Furthermore, although many people derive benefit from antidepressant medication, approximately one in three will experience another depressive episode within six months of antidepressant discontinuation [5]. An initially successful treatment is therefore still too often followed by a relapse.

Randomized controlled trials indicate that continual maintenance treatment with antidepressant medication reduces the risk of relapse or recurrence [5–8]. Nevertheless, maintenance treatment does not completely eliminate the risk of suffering from breakthrough depression while still on treatment, or from further depressive episodes after subsequent discontinuation [9]. Additionally, many people experience unpleasant side effects of antidepressant medication, such as weight gain and sexual dysfunction [10]. Thus, not all individuals who experience a depressive episode benefit equally from continuing medication after achieving remission. There is therefore a pressing clinical need to distinguish those who can safely discontinue antidepressants from those with a higher risk of relapse following discontinuation.

Current clinical guidelines recommend continued treatment for at least six months after obtaining remission from a first episode of depression, and at least two years of treatment after remission for patients deemed to be at high risk of relapse [11, 12]. The risk of relapse is assessed using one or more of different predictors, such as the number of prior episodes [11], physical and psychological comorbidities [11], ethnicity [13], a melancholic subtype [14], anxiety [15], somatic pain [16], and previous response to medication [17]. However, several of these predictors lack robust replication studies to support their relevance (for a review see [18]). Where replications do exist, these sometimes reach conflicting conclusions, for example regarding the effect of the number of previous episodes on future relapse risk [6, 19]. Other predictors are difficult to reliably measure; for example, in clinical practice, previous response to treatment is often unclear [18]. This uncertainty not only calls for continued investigation into existing markers of relapse, but also motivates a search for novel relapse predictors.

In this study we evaluate delay discounting, which is thought to quantify a person’s impatience to receive reward, as a candidate behavioural predictor of depressive relapse following antidepressant discontinuation^1^. Delay discounting can be quickly assessed, by offering participants a series of choices between immediate and delayed rewards of varying magnitude. Conventionally, such choices are used to estimate a parameter termed the ‘discount rate’, which captures how steeply the subjective value of reward decreases as it is delayed. Higher discount rates imply a steeper decrease in reward value with delay, and thereby greater impatience. The behavioral and neural correlates of delay discounting have been extensively studied (see e.g. [20–23]).

Existing evidence suggests that delay discounting is a plausible candidate marker of depressive relapse following antidepressant discontinuation. Firstly, studies have reported higher delay discount rates amongst people with Major Depressive Disorder (MDD) when compared to healthy controls [24–27]. Notably differences in discounting between depressed participants and non-depressed controls are not found reliably across all studies and comparisons. Some studies find no significant difference [28, 29], while others find differences from healthy controls only amongst sub-groups of depressed participants [24, 27], or only for larger rewards [26]. Nevertheless, a meta-analysis of seven case-control studies supports a conclusion of elevated discounting in MDD, with a small effect size (Hedges *g* = 0.38) [30]. The greater impatience observed in MDD has been interpreted as resulting from the pessimistic future outlook which is a feature of depression [4, 31–36] and as reflecting the loss of evaluative differentiation concerning future outcomes [37].

Secondly, most antidepressant medications are believed to increase serotonin levels, which is thought to be crucial for their therapeutic effect [38, 39], while discounting is also found to be sensitive to serotonergic manipulations. Tryptophan depletion, which lowers brain serotonin levels, induces acute symptomatic relapse in patients with remitted depression [40, 41], and has been found to increase discount rates in healthy participants [42, 43] (though Tanaka et al. did not replicate this effect) [44]. Furthermore, a small study found that discount rates were reduced by acute administration of a selective serotonin reuptake inhibitor amongst participants with Attention Deficit Hyperactivity Disorder [45]. More definitively, rodent studies have demonstrated that stimulating serotonergic neurons in the dorsal raphe, or their projections to medial prefrontal cortex, augments an animal’s willingness to wait for reward [46, 47], while lesioning or blocking serotonergic neurotransmission increases impatience [48–50].

In summary, evidence indicates that discounting is increased in MDD, increases following serotonin depletion and decreases following enhancement of serotonin release. Thus, delay discounting is a candidate marker of both serotonergic function and depressive cognition. Based on these findings, our primary hypothesis was that patients with remitted MDD who show higher delay discounting are at increased risk of relapse following antidepressant discontinuation. A secondary hypothesis was that antidepressant discontinuation results in an increase in delay discounting, and that the magnitude of this early increase in discounting predicts subsequent depressive relapse. We tested these hypotheses within the AIDA (Antidepressiva Absetzstudie) study – a two-center, longitudinal, observational study of antidepressant discontinuation [51–53]. We also tested how delay discounting is related to depression symptom scores and other psychometric data amongst this sample of patients with remitted depression.

## Methods and materials

### Participants and study design

Data from the AIDA study has been analysed previously [51–53]. However, the delay discounting data reported here have not previously been examined. The dataset consists of: i) participants treated with antidepressant medication (ADM), who decided to discontinue their antidepressant medication independently from study participation, after being diagnosed with Major Depressive Disorder, and ii) healthy control (HC) participants matched for age, sex and education to the ADM group. Healthy controls were excluded if there was a lifetime history of DSM IV Axis I or Axis II disorders, with the sole exception of nicotine dependence. Recruitment criteria for the ADM group included: (a) at least one severe [54] or multiple depressive episodes, (b) initiation of antidepressant treatment during the last depressive episode, and (c) achieving stable remission, assessed by a score of less than 7 on the Hamilton Depression Rating Scale 17 [55] for 30 days. See [51–53] for detailed inclusion and exclusion criteria.

All participants gave informed written consent and received monetary compensation for their time. Ethical approval for the study was obtained from the cantonal ethics commission Zurich (BASEC: PB_2016-0.01032; KEK-ZH: 2014-0355) and the ethics commission at the Campus Charité-Mitte (EA 1/142/14), and procedures were carried out in accordance with the Declaration of Helsinki.

**Fig. 1:**
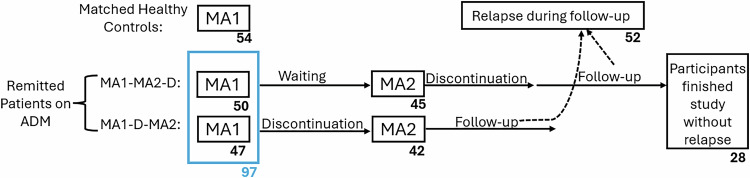
Study Design. We recruited remitted patients treated with antidepressant medication (ADM) and healthy controls matched for age, sex and education to the patients group. Patients were assessed at Main Assessment 1 (MA1) to identify features characterizing the remitted, medicated state. Next, patients were randomized to either discontinue their medication before MA2 (bottom arm, “group MA-1-D-MA2” or enter a waiting period while continuing their ADM, matched to the length of discontinuation time (top arm, “group MA1-MA2-D”). Discounting was assessed at MA1 and MA2, to investigate the effects of discontinuation. Patients in the MA1-MA2-D discontinued their ADM after MA2. After discontinuation, all patients entered the follow-up (FU) period of 6 months, during which some patients relapsed. Numbers below each box indicate the number of subjects in that group. The numbers below each box indicate the number of subjects in the corresponding group.

As shown in Fig. 1, participants were assessed and compared at Main Assessment 1 (MA1) to identify features characterising the remitted, medicated state. Next, patients were randomised to either discontinue their medication at MA1 (MA1-D-MA2) or enter a waiting period approximately matched to the length of discontinuation time (group MA1-MA2-D). Patients in the waiting group discontinued their ADM after Main Assessment 2 (MA2). Details of the randomisation procedure are provided in the Supporting Material. After discontinuation, all patients entered a six month follow-up (FU) period, wherein some patients experienced a relapse.

The data analysis plan for the current study was preregistered [56], and is provided in Supplementary Table 1. All participants answered rating questionnaires, among which, the measures of prior interest for the present study were Hamilton Depression Scale (HAM-D), Emotion Regulation Questionnaire (ERQ), Brief Self-Control Scale (BSCS), Daily Hassles, Satisfaction with Life Scale (SWLS), Adverse Childhood Experience (ACE), Childhood Trauma Questionnaire (CTQ), Traumatic Life Events Questionnaire (TLEQ), and the Mehrfachwahl-Wortschatz-Intelligenztest (MWT-B).

We also conducted a power analysis for group differences prior to the study commencement. The description of which is provided in the Supporting Material.

### Delay discounting tasks

Delay-discounting procedures estimate the indifference point at which a smaller but immediately available reward, *r*, and a larger but delayed reward, *R*, have approximately the same subjective value to the participant. Here, participants completed two delay discounting tasks to estimate indifference points for rewards across a range of delays. The first task was Kirby’s monetary choice questionnaire (MCQ) [57], which consists of 27 items each asking participants to choose between an immediate and a delayed reward. In the second task, participants answered an adaptive version of the questionnaire [58], wherein a discount rate is estimated after each choice the subject makes, and the next immediate and delayed rewards offered are provided from the currently estimated indifference point. At each step, this procedure elicits the most informative choice, based on a participant’s estimated discount rate. The procedure continues until a stable estimation of the indifference point is reached [58]. Including two tasks, rather than one, was intended to bolster reliability.

In this study, rewards were hypothetical. Although one previous study found a small reduction in discount rates for real as opposed to hypothetical rewards [59], a number of other studies report no systematic differences in discounting for real and hypothetical rewards [60–62], suggesting that assessing discounting for hypothetical rewards is a valid procedure.

### Delay discounting model and model fitting procedure

We modeled the participants’ choices using a standard hyperbolic model [63]:

1 $$V\left(R,d\right)=\frac{R}{1+{Kd}}.$$

**Fig. 2:**
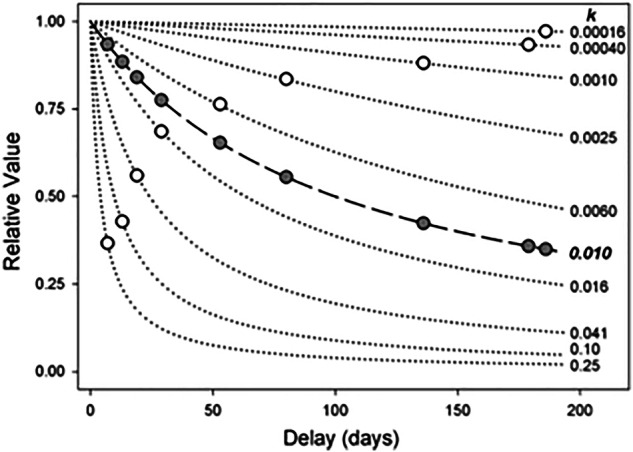
Hyperbolic delay discounting. Illustration of the hyperbolic model of delay discounting for a subset of nine questions from Kirby’s monetary choice questionnaire (MCQ) consisting of small amount of delayed reward (25$–35$). Each open white circle represents one of the nine questions: its X-coordinate indicates how long one would have to wait for the delayed reward (delay, d), while its Y-coordinate indicates the value of the immediate, no delay reward relative to the delayed reward (relative value, V/R). Each dotted curve represents the hyperbolic delay discount rate, K, at which a participant would be indifferent between immediate and delayed rewards for each specific choice. The dashed curve corresponds to a discount function with *K* = 0.01. A person with this fitted value of the discount rate would choose the immediate rewards in the questions with $$K$$ values larger than $$0.01$$ (bottom four hyperbolic curves), and would choose the delayed reward on the questions with $$K$$ values smaller than $$0.01$$ (top five hyperbolic curves). Open grey circles represent the subjective values, V(R, d), predicted by the dashed curve for each value of delay (d) of the nine questions. Adapted from [49, 59, 71].

This equation describes the subjective value, *V*, of a reward, *R*, available after a delay *d*. $K \,{{{\rm{is}}}}\; {{{\rm{a}}}}\; {{{\rm{discount}}}}\; {{{\rm{rate}}}},{{{\rm{estimated}}}}\; {{{\rm{from}}}}\; {{{\rm{participants}}}}{{\hbox{'}}}{{{\rm{indifference}}}}\; {{{\rm{points}}}}.\;{{{\rm{Higher}}}}\; {{{\rm{values}}}}\; {{{\rm{of}}}}$ *K* reflect greater impatience and reduced tolerance for delay [52]. The hyperbolic model of delay discounting is illustrated in Fig. 2. A generalization of this hyperbolic model includes an exponent on the delay term, which adjusts the curvature of the discount curve [64, 65]. Here, since we are interested in individual differences, we omit this exponent in favour of the standard hyperbola, which captures variability in discounting with a single parameter, *K*.

We fitted the delay discounting model using a Bayesian hierarchical (mixed-effects) logistic regression [66]. This general procedure is widely used to fit parameters in decision making tasks, see e.g. [66–69]. In brief, estimated discount rate yields a difference in subjective value between immediate and delayed rewards for each choice. A logistic sigmoid (softmax) function, $\sigma \left(x\right)=\frac{1}{1+\exp (-x)}$, transforms this subjective value difference into a probability of choosing the immediate reward on each choice. We used an optimization procedure to find parameters that maximize the joint probability of each participant’s observed choices, assuming an empirical prior distribution over discount rates. This prior distribution, which is estimated using Expectation-Maximization (EM), served to regularise the inference and prevent parameters that are not well-constrained from taking on extreme values [66]. The reader is referred to [66] for the full technical details of the routine.

To maximize reliability, we fitted the model to the concatenated answers of both the classic and the adaptive versions of the questionnaires. We estimated the goodness-of-fit of the resulting model using McFadden’s pseudo-R², averaged across all subjects [70]. Furthermore, after fitting the model, we excluded subjects whose model accuracy is not significantly better than chance, estimated by a corresponding binomial test with a significance threshold of 0.05. Specifically, we calculated  $$p={\sum }_{i=k}^{n}\left(\begin{array}{c}n\\ i\end{array}\right){0.5}^{i}{0.5}^{n-i} $$  and excluded participants for which *p* > 0.05.  Here, *n* is the number of questions in the questionnaire, *k* is the number of correctly classified answers and *p* denotes the p-value of a right-tailed binomial test, i.e., the probability of obtaining *k* or more correct classifications out of *n* by chance.

### Data analysis

Analyses were performed in Matlab (R2023a) according to the pre-registered analysis plan provided in Supplementary Table 1 and also in [56]. In each analysis step reported below, we refer the reader to the corresponding analysis step from Supplementary Table 1, or indicate the step was not part of the original analysis plan. In this study we report analyses of discounting choice data. For the sake of clarity, we divide the analyses into three categories: i) prediction of relapse, ii) effect of discontinuation, and iii) discounting *in remitted MDD*.

Since previous studies show that discount rates, *K*, are log-normally distributed [71, 72] we test for differences in *log K* rather than *K*. Unless otherwise stated, paired and independent-samples *t*-tests were used to compare group means. Given that each group comprised at least 30 participants, the Central Limit Theorem supports the assumption that the sampling distribution of the mean was approximately normal. Indeed, for each comparison, normality and equal variance were tested using Kolmogorov-Smirnov (MATLAB *kstest* function) and Bartlett tests (MATLAB *vartestn* test), respectively. For the few comparisons where either test rejected the null hypothesis of normality or equal variance, a non-parametric test was used: Wilcoxon Signed Rank for paired samples or Rank Sum for independent samples. We report means and standard deviations (or, where relevant, medians and interquartile ranges) for all comparisons in Supplementary Table 5.

### Prediction of relapse

We started by testing for association between discount rate and relapse by using a one-tailed two-sample *t*-test to test if *log K* at MA1 was greater in patients who relapsed than in patients who did not relapse during follow-up. This test explores the potential of baseline *log K* as a predictor of future relapse. We also used a one-tailed two-sample t-test to test if the *change* in *log K* between MA1 and MA2 (gain scores) differed between subjects from the MA1-D-MA2 group who relapsed during follow-up and subjects from the MA1-D-MA2 group who did not relapse during follow-up. This test examines whether a change in *log K* following discontinuation is associated with subsequent relapse. The tests detailed in this paragraph were not part of the pre-registered analysis plan.

In addition, to test for an association between time to relapse and discount rate, we used MATLAB *coxphfit* function to fit a Cox proportional hazards model with days to relapse as the dependent variable. We fitted two such models, with independent variables as i) *log K* at MA1 (Step (4) in the analysis plan), or ii) *log K* at both MA1 and MA2 (Step (5) in the analysis plan).

To examine whether discount rates can predict subsequent relapse, we fitted a logistic regression model with an L1 regularization (known as “Lasso” [73]), as implemented by the *lassoglm* function in Matlab, with relapse as the dependent variable and either *log K* at MA1 (Step (4) in the analysis plan), or both *log K* at MA1 and *log K* at MA2 as independent variables (Step (5) in the analysis plan). Consistent with the analysis plan, the model was trained on subjects from the Zurich sample, with a view to testing on the Berlin sample. We applied tenfold cross validation with stratification to optimize the value of the L1-regularization parameter.

### Effect of discontinuation

We also hypothesized that discontinuation at MA1 would be associated with an increase in *log K* (between MA1 and MA2), assessed relative to the group who discontinued at MA2. To test for this, we fitted a linear mixed effects model using MATLAB *fitlme* function, with *log K* at both timepoints as the dependent variable, and group (i.e., MA1-D-MA2 or MA1-MA2-D), timepoint (i.e., MA1 or MA2) and [group × timepoint], as independent (fixed effect) variables (Step (2) in the analysis plan). We included a random slope term for each participant.

### Discounting in remitted MDD

We used a one-tailed two-sample t-test to test the hypothesis that *log K* at MA1 was greater in patients than in controls (Step (1) in the analysis plan). We also tested for associations between *log K* at MA1 and scores on the various rating scales, using simple linear regression, with *log K* as the dependent variable. Additionally, we expressed pairwise associations between *log K* at MA1 and each rating scale as a Spearman correlation coefficient (Step (3) in the analysis plan).

Finally, we tested whether *log K* at MA1 was associated with a change in depression (HAM-D) scores over time, independent of discontinuation (Step (6) in the analysis plan). To do so, we fitted a linear mixed effect model, wherein the dependent variable is HAM-D score (at MA1 or MA2), the independent variables (with fixed effects) are *log K* at MA1, timepoint (MA1 or MA2), a [*log K*_MA1_ × timepoint] interaction, discontinuation group (MA1-D-MA2 vs. MA1-MA2-D) and [discontinuation group × timepoint] interaction. We included a random slope for each participant. Here, the [*log K*_MA1_ × timepoint] interaction term expresses the extent to which a change in depression score across time depends on *log K* at baseline, whereas the [discontinuation group × timepoint] interaction term controls for possible confounding that results from testing on two discontinuation groups that differ in the time of withdrawal. Here we hypothesized that participants with higher baseline discounting would show less improvement in depressive symptoms across time.

Complementary analysis methods and results that appear in the a priori analysis plan are provided in the Supplementary Material. As set out in the analysis plan, all comparisons were performed first on the Zurich sample, with a view to testing on the Berlin sample as an out-of-sample validation of predictive accuracy. However, where no significant associations between *log K* and the variables of interest were found in either sample, we pooled both samples to maximize power. We report these pooled analyses here.

## Results

### Sample description

Out of 104 patients with remitted MDD and 57 controls who were initially recruited, 97 patients (71 from Zurich and 26 from Berlin; 77% female, average age 34.78) and 54 controls (32 from Zurich and 22 from Berlin; 70% female, average age 33.52) answered the discounting questionnaire at MA1. 47 and 50 patients were randomized at MA1 to the discontinuation (MA1-D-MA2) and continuation group (MA1-MA2-D), respectively. 10 patients dropped out before MA2 and 7 more patients dropped during the follow-up period. The 17 dropouts were excluded from the prediction of relapse analysis. Among the included patients, 52 remained well (65%) and 28 (35%) relapsed during the follow-up period. The numbers of participants in each group are also indicated in Fig. 1. At baseline, HAM-D scores in the remitted patient group, although below the clinical threshold for MDD, were significantly higher than those in the control group (HAM-D controls mean = 0.38, median = 0, HAM-D patients mean = 1.81, median = 1; two-sample, two-tailed t-test *t*(147) = 5.15, *p* < 0.001; Wilcoxon rank sum test p < 0.001).

### Model fitting

Model accuracy met the (binomial test) accuracy criterion described above for all participants, and therefore no participants were excluded. The average model accuracy was 85%; mean McFadden’s pseudo-${R}^{2}$ across subjects was 0.59, indicating a good fit to the data. Discount rates obtained from the adaptive discounting questionnaire and the Kirby MCQ were only moderately correlated (Spearman ρ = 0.32, *p* < 0.001).

In addition, no significant associations were found between *log* K at baseline and any possible confounding factors tested. The details and results are provided in Supplementary Table 2.

### Prediction of relapse

**Fig. 3:**
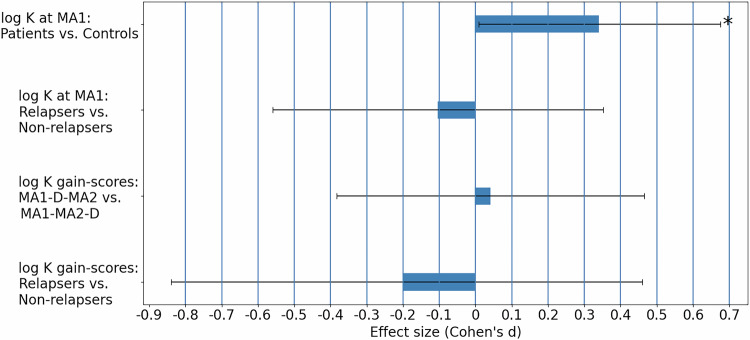
Effect sizes for group differences in *log K.* Cohen’s d effect size, for various group comparisons. The top bar shows the comparison of log K at MA1 between controls and patients, where the effect size in this case indicates that the average of log K in the Patients group (at both sites) at MA1 is greater than the average of log K in the Controls group at MA1. The second bar from above shows the comparison of log K at MA1 between patients who subsequently relapsed and patients who did not, where the effect size in this case indicates that the average of log K in non-relapsers at MA1 is greater than the average of log K in relapsers at MA1. The third bar shows the comparison of the change in log K between the two timepoints (gain scores), between patients who discontinued their treatment at MA1 (MA1-D-MA2) and patients who continued their treatment until MA2 (MA1-MA2-D), where the effect size indicates that the average of gain scores in the MA1-D-MA2 group is greater than the average of gain scores in the MA1-MA2-D group. The bottom bar shows the comparison of the change in log K between the two timepoints (gain scores), between patients from the MA1-D-MA2 group who subsequently relapsed and patients from the MA1-D-MA2 group who did not, where the effect size indicates that the average of gain scores in the non-relapsers group was greater than the average of gain scores in the relapsers group. Error bars represent 95% confidence interval for Cohen’s d effect size, estimated using MATLAB meanEffectSize function. Group difference p-value: * 0.01<p < 0.05.

We found no significant difference in *log K* at MA1 between subjects treated with ADM who relapsed during follow-up and subjects treated with ADM who did not relapse (*t*(78) = 0.44, *p* > 0.25,two-tailed two-sample; Cohen’s *d* = 0.10). Furthermore, a change in *log K* following discontinuation (i.e., between MA1 and MA2 amongst the MA1-D-MA2 group), did not differ significantly between participants who subsequently relapsed and those who did not relapse (*t*(37) = 0.58, *p* > 0.25, one-tailed; Cohen’s *d* = 0.20), see Fig. 3. In a Cox proportional hazards regression model, including *log K* at both timepoints, neither *log K*_MA1_ nor *log K*_MA2_ were significantly associated with days-to-relapse (Coefficient *log K*_MA1_ = −0.02, *p* > 0.25; coefficient *log K*_MA2_ = −0.08, *p* > 0.25), nor was *log K*_MA1_ associated with relapse when entered into a separate regression model (Coefficient = −0.08, *p* > 0.25).

In the prediction of relapse, the regularized regression weights were found to be all zero, resulting in a balanced accuracy of 0.5 and reflecting the balanced proportion of the majority class. For the sake of completeness, the distribution of baseline *log K* in the different relapse groups is shown in Supplementary Figure S1.

### Effect of discontinuation

Contrary to our secondary hypothesis, antidepressant discontinuation was not associated with a significant increase in impulsive choice, relative to continuing medication. Specifically, in a linear mixed effects model with *log K* as the dependent variable, we found no significant [timepoint × discontinuation group] interaction (${\beta }_{{timepoint\; x\; group}}$ = 0.04, *t*(180) = 0.15, *p* > 0.25). In other words, discontinuation did not significantly alter a change in *log K* across time. Main effects of timepoint and group were also small and non-significant (${\beta }_{{group}}$ = −0.05, *t*(180) = −0.10, *p* > 0.25 ; ${\beta }_{{timepoint}}$ = 0.10, *t*(180) = 0.56, *p* > 0.25).

We further explored this null finding in a *post hoc* analysis, by performing a one-tailed two-sample *t*-test on *log* K gain scores to test whether *log K* increased more in patients who discontinued at MA1 (MA1-D-MA2) than in patients who discontinued at MA2 (MA1-MA2-D). To prevent error accumulation due to the additivity of noise, in model fitting, the difference between *log K* at MA1 and *log K* at MA2 was estimated concurrently with *log K* at MA1. Again, we found no significant difference in the change in *log K* between MA1 and MA2, among the MA1-D-MA2 group compared with the MA1-MA2-D group (t(82) = 0.19, *p* > 0.25; Cohen’s *d* = 0.04), also indicated in Fig. 3.

A possible explanation for these null results would be that our delay discounting measure was unreliable. If this were the case, we would expect no consistent relationship between discounting at MA1 and MA2. Contrary to this idea however, across all patients we found a moderate correlation between *log K* at the two timepoints (*r* = 0.72, *p* < 0.001). Similar test-retest correlations were observed in both the MA1-D-MA2 (*r* = 0.80, *p* < 0.001) and MA1-MA2-D groups (*r* = 0.65, *p* < 0.001). These results indicate that the rank order of discounting across participants was moderately stable over time, supporting the reliability of our discounting measure.

A further possible explanation for observing no effect of discontinuation on impulsivity would be that discontinuation produced no significant withdrawal syndrome in the study participants. Against this, the MA1-D-MA2 group exhibited a statistically significant increase in depressive symptoms following discontinuation (MA1 HAM-D mean = 1.65, median = 1; MA2 HAM-D mean = 3.16, median = 3; *t*(39) = 4.39, *p* < 0.001, two-tailed; Wilcoxon signed rank *p* < 0.001). No such symptom change was observed in the MA1-MA2-D group, who did not discontinue medication until after the second timepoint (MA1 HAM-D mean = 1.98, median = 2; MA2 HAM-D mean = 2.18, median = 2; *t*(40) = 0.34, *p* = 0.733, two-tailed; Wilcoxon signed rank *p* = 0.610). Furthermore, symptom change between the two timepoints in the MA1-D-MA2 group was significantly greater than that in the MA1-MA2-D group (two-sample t-test, *t*(79) = 2.77, *p* = 0.007, two-tailed; Wilcoxon rank sum *p* = 0.013). These findings indicate a detectable effect of discontinuation.

### Discounting in remitted MDD

Group comparison of *log K* between healthy controls and patients with remitted MDD (treated with ADM) at MA1 revealed significantly higher discount rates in the patient group (*t*(149) = 2.03 and *p* = 0.022, one-tailed; Cohen’s *d* = 0.34), also indicated in Fig. 3. Notably both groups showed low levels of impulsivity, and the absolute difference in *K* between the two groups was small. Mean K in the remitted MDD group was 0.0065, corresponding to indifference between a reward of 75 euros received in 20 days and an immediate reward of 66 euros. Mean K in the control group was 0.0037, corresponding to indifference between a reward of 75 euros received in 20 days and an immediate reward of 70 euros.

**Fig. 4:**
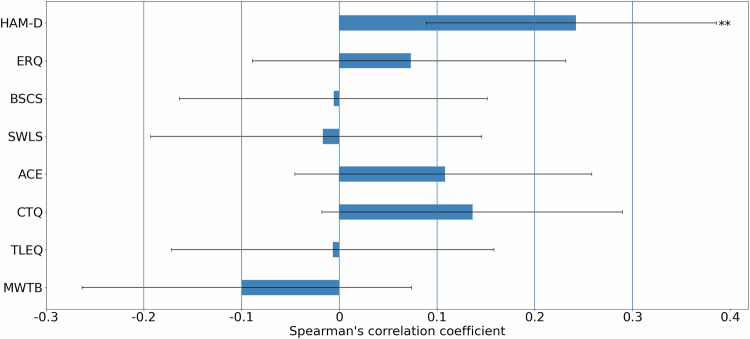
Correlations between *log K* at MA1 and rating scales. HAM-D Hamilton Depression Scale, ERQ Emotion Regulation Questionnaire (ERQ), BSCS Brief Self-Control Scale, SWLS Satisfaction with Life Scale, ACE Adverse Childhood Experience, CTQ Childhood Trauma Questionnaire, TLEQ Traumatic Life Events Questionnaire, MWTB Mehrfachwahl-Wortschatz-Intelligenztest. Error bars represent 95% confidence interval for Spearman’s correlation coefficient estimated using 10,000 bootstrap iterations. Group difference p-value: * 0.01<p < 0.05, ** 0.001<p < 0.01.

As shown in Fig. 4, depressive symptoms (measured by the HAM-D scale) were significantly correlated with baseline discount rate, *log K*_MA1_ (Spearman ρ = 0.24, *p* = 0.003), an association which survived Bonferroni correction for multiple comparisons (*p* = 0.022, corrected for 8 comparisons), and was also present when testing only on the patients’ group (Spearman ρ = 0.23, *p* = 0.025). Other questionnaire instruments did not exhibit significant correlations with *log K*_MA1_ (Fig. 4). We note that baseline discount rate showed a significant correlation with two *subscales* of the CTQ questionnaire, namely CTQ-physical abuse (Spearman ρ = 0.18, *p* = 0.023) and CTQ-emotional neglect (Spearman ρ = 0.16, *p* = 0.049). See Supplementary Figure S2 for the comparisons with other questionnaire subscales. When all questionnaire variables were entered into a linear regression model with *log K*_MA1_ as the dependent variable, only HAM-D emerged as a significant explanatory variable (coefficient estimate = 0.19, *t*(142) = 2.51, *p* = 0.013). Coefficients and *t*-statistics for the remaining rating scales are provided in Supplementary Table 3.

We went on to test for an association between a change in depression across time, and baseline discounting (at MA1), in a mixed-effects linear regression with HAM-D scores as the dependent variable. We found a significant main effect of *log K*_MA1_ (coefficient estimate = 0.56, *t*(150) = 2.26, *p* = 0.025). This result is consistent with the findings reported above of a correlation between *log K*_MA1_ and HAM-D at MA1. We found no significant main effect of timepoint (coefficient estimate = 0.01, *t*(150) = 0.01, *p* = 0.989); here, the positive coefficient indicates that the average participant showed a marginal, albeit non-significant, increase in HAM-D score across time. There was a significant [timepoint × *log K*] interaction (coefficient estimate = −0.30, *t*(150) = −2.01, *p* = 0.045). Here, contrary to our prediction, the negative coefficient indicates that participants who were more impulsive (higher *log K*) at baseline showed a greater reduction in depression score across time. We found no significant effect of discontinuation group(coefficient estimate = 0.19, t(150) = 0.22, p = 0.820), nor a significant effect of the [discontinuation group × timepoint] interaction (coefficient estimate = −0.47, t(150) = −0.94, p = 0.345).

## Discussion

In this pre-registered analysis, we examined the potential of delay discounting as a behavioral marker of relapse after antidepressant discontinuation. There is a priori evidence to suggest that delay discounting might help predict illness trajectory following discontinuation of antidepressant medication (ADM). To the best of our knowledge, the present study is the first to prospectively examine i) whether discounting predicts future depressive relapse following ADM discontinuation, and ii) the effect of ADM discontinuation on delay discounting. Our results suggest that delay discounting is not altered by ADM discontinuation to a clinically meaningful extent. Furthermore, we found that neither baseline delay discounting, nor a change in discounting following ADM discontinuation were predictive of future depressive relapse. However, we did find significantly steeper delay discounting amongst patients with remitted MDD, compared with controls (Cohen’s *d* = 0.34), and a robust relationship between the discount rate and depressive symptoms (Spearman ρ = 0.24).

We note that our observed correlation between delay discounting and depressive symptoms may be attributable to subdomains of depressive symptoms. This possibility accords with previous studies finding relationships between discounting and symptom variables such as hopelessness, anhedonia [31] and suicidal ideation [24] or acts [35]. Owen et al. [37] reported a loss of evaluative differentiation concerning future outcomes in patients with MDD, which might serve as an explanation for our findings.

To our knowledge, the present study is the first to find significantly elevated delay discounting amongst medicated patients with remitted MDD. This finding is consistent with previous studies that have observed a relationship between trait-level impulsivity (e.g., as assessed in self-report rating scales) and remitted depression [74, 75]. A previous study by Pulcu et al. [26], which compared delay discounting amongst people with remitted, medication-free MDD and healthy controls, found that patients with remitted depression showed marginally steeper discounting than controls, however this difference was statistically significant only for larger rewards. In both the study of Pulcu et al., and the present study, depressive symptoms were significantly correlated with discount rate across all participants. The elevated discounting seen here in remitted MDD might therefore reflect residual, sub-clinical depressive symptoms. In keeping with this hypothesis, the remitted patient group exhibited higher depressive symptom scores than the control group.

Alternatively, discounting might partly capture a trait-level vulnerability to depression, which persists despite symptom resolution. Previous studies find that delay discounting indeed has properties of a trait variable, being conserved across different types of reward [76], with moderate test-retest reliability [77]. Our combined delay-discounting score exhibited a similar degree of stability within-participants across the two time points of the study (*r* = 0.72) to that recently reported in meta-analysis (*r*  =  0.670, 95% CI [0.618, 0.716]) [77]. These findings suggest that delay discounting can be considered a trait variable. However, since our study did not measure discounting longitudinally in patients as they moved into remission, we have no direct evidence to support an hypothesis that steeper discounting is a vulnerability factor for MDD.

In the current study, higher discounting at baseline was not predictive of future relapse following discontinuation; nor was baseline discounting associated with worsening depressive symptoms between the two timepoints of the study (up to six months apart). The relatively small sample size of this study may be underpowered to detect subtle relationships. For example, a post-hoc power analysis for a two-tailed two-sample t-test with a type I error rate of α = 0.05, comparing 28 relapsers and 52 non-relapsers, indicates a power of 0.8 to detect an effect size of *d* = 0.66 and a power of 0.95 to detect an effect size of *d* = 0.85. Furthermore, the power to detect a medium effect size of *d* = 0.5 is 0.55 (using G^★^-power 3.1 [78]). While we do not provide evidence for the absence of effects, power considerations inform our interpretation and suggest that large effect sizes, greater than 0.5, are unlikely.

Another limitation that restricts our ability to accurately predict future risk of relapse is the limited six-month follow-up period, which may lead us to overlook patients who did not relapse during this time period but might have relapsed if observed for a longer duration. Nevertheless, our null finding suggests that, if discounting is indeed a trait-level vulnerability factor for MDD, this effect is too small to be clinically meaningful over short-term follow up.

A potential limitation of our statistical analyses concerns how the hierarchical Bayesian procedure used to estimate the discount rate was applied in the context of a regularized regression analysis to predict relapse. Within the cross-validation framework used to optimize the regularization parameter discount rate was not fitted separately for training and validation sets of each fold, resulting in non-independent estimates between these two sets, and potentially optimistic estimates of prediction accuracy. However, this concern is mitigated in our specific case as the prediction results remain insignificant. Furthermore, the issue does not affect the critical test of training the model on the Zurich dataset and testing it on the Berlin dataset.

Contrary to our prediction, higher impulsivity at baseline was associated with a marginally significant *decrease* in depressive score across time. We are uncertain as to the explanation for this effect. We speculate that higher impulsivity is linked to greater venturesomeness, which encourages exploration and thereby recovery from depression. Alternatively, this unexpected finding might arise due to regression to the mean of depressive symptoms across time. That is, since participants with higher baseline impulsivity tended to show higher baseline depressive symptoms, if higher baseline symptoms tended to regress to the mean over time, impulsivity also would appear to be weakly associated with symptomatic improvement. However, since this finding is against our prior predictions, further replication is needed.

A secondary hypothesis was based on an idea that discounting would be a sensitive marker of psycho-physiological changes following ADM discontinuation. However, we did not observe an increase in impulsivity following ADM discontinuation. Specifically, we did not find a significant change in *log K* amongst remitted patients who discontinued their treatment (MA1-D-MA2 group), relative to remitted patients who continued their treatment (MA1-MA2-D group). This finding is also consistent with an AIDA study of effort-reward tradeoffs [52], where the authors found no effect of ADM discontinuation on choices of high-effort high-reward options. Although our finding may be the result of limited statistical power, the relatively small effect sizes obtained from the corresponding group comparisons (Cohen’s *d* < 0.1), as well as the significant group differences obtained in other comparisons, suggest otherwise. Indeed, our finding of a small yet statistically significant increase in depressive symptoms following ADM discontinuation, indicating that stopping medication had a clinically detectable effect, further points to a dissociation between discounting and discontinuation.

We had hypothesized that discounting might be sensitive to decreases in serotonergic neuromodulation following antidepressant discontinuation. However, although discounting has been shown to be sensitive to serotonergic manipulations, it is unclear whether the elevated discounting observed in MDD is linked to changes in serotonin. Furthermore, the directionality and temporality of the adaptive changes in the 5-HT system following antidepressant discontinuation are uncertain. Some evidence points to a reduction in the extracellular 5-HT levels following discontinuation [79, 80], while other studies indicate a rebound above pre-treatment levels (see e.g. [80–83]). Taking these considerations together, a lack of association between discounting and ADM discontinuation is not out of keeping with the state of existing knowledge concerning causal relationships between serotonergic function, depressive disorders and ADM.

## Conclusion

Delay discounting is not strongly affected by ADM discontinuation and therefore appears to be of limited use as a biomarker for decisions related to anti-depressant discontinuation.

## Supplementary information

Supplementary Material

## Data Availability

The datasets generated and analysed during the current study are available from the corresponding author on reasonable request and in line with the ethical rules.

## Footnotes

1 The term ‘relapse’ is conventionally reserved for episodes within the first six months, after which the term ‘recurrence’ is usually employed. In this study, since the time from first episode varied amongst our patient group, we do not distinguish between relapse and recurrence, and adopt the term ‘relapse’ throughout.

## Code availability

The MATLAB code used to analyse the data is available upon reasonable request to the corresponding authors.
