# Supplementary material for: Delay discounting correlates with depression but does not predict relapse after antidepressant discontinuation
Source: Mol Psychiatry. 2026 Jan 8;31(5):2445–53. doi: 10.1038/s41380-025-03402-5 (PMC13099390; doi:10.1038/s41380-025-03402-5)
Supplement: Supplementary file 1 — Supplementary Material [file 41380_2025_3402_MOESM1_ESM.docx]

Delay discounting correlates with depression but does not predict relapse after antidepressant discontinuation

**Supporting Material Online**

**Randomisation Procedure**

We assumed a priori that severity of a person's depressive illness might affect relapse rates. This hypothesis was tested in a separate study^51^. Hence, to ensure that both groups would contain a sample with equal distributions of severity, we stratified patients into a severe and a non-severe group. If patients fulfilled either of the following criteria, they were assigned to the severe group: 1) more than three prior episodes, 2) more than seven depressive symptoms during the last episode, 3) severely impaired social functions, i.e. strongly isolated, disabled or aggressive, 4) engagement in activities almost nonexistent, 5) capacity to work almost nonexistent. We, additionally, stratified for site. Group membership was allocated by a randomization algorithm. The first ten subjects at each site were not randomized, but all assigned to the MA1-D-MA2 arm.

**Lasso regression modeling with *log K* at baseline as dependent variable and depression rating questionnaire scores as independent variables**

To further examine whether depression rating questionnaires can predict discounting, we used a regression model with both an L1 regularization, as implemented by the lassoglm function in Matlab, with log K at baseline as the dependent variable and scores to depression rating questionnaires as independent variables. We applied a tenfold cross validation to optimize strength of the L1-regularisation parameter. This was repeated for 100 equally spaced regularization parameter values ranging from $3.5\times{10}^{-5}$ to 0.35, and the parameter that resulted in the minimum cross-validated mean squared error was chosen.

The predictors having non-zero coefficients were HAM-D (coefficient estimate=0.126), CTQ (coefficient estimate=0.002) and MWTB (-0.034), and the MSE was 2.6812. Predicted baseline log K were found to be moderately correlated with true baseline log K (Spearman’s $\rho$=0.303, p<0.001).

Following the a priori analysis plan, we also carried out similar analysis in which the model was trained on log K values of subjects from the Zurich sample, and then tested on log K values estimated of subjects from the Berlin sample, serving as a hold out. The predictors having non-zero coefficients were HAM-D (coefficient estimate=0.075) and MWTB (-0.041). The MSE on the whole training (Zurich) sample was 2.718, and the MSE on the whole test (Berlin) sample was 2.322. Predicted baseline values of log K in the test sample were correlated with true baseline log K in the test sample, however this correlation did not reach statistical significance (Spearman’s $\rho$=0.219, p=0.1337).

***A priori* power analysis**

The *a priori* power analysis reported here was conducted prior to the original AIDA study^^[[1]](#footnote-1)^^ to estimate the required sample size for detecting a group difference (i.e. a t-test). We assumed an effect size of $d=1.2$and an attrition rate of 20% over the six-month follow-up period. Some patients may have re-entered therapy without meeting the criteria for a full relapse, forming an intermediate group. We assumed that 30% of relapse patients would fall into this category.

Six months relapse rates on placebo ranges from 34-62%^^[[2]](#footnote-2)^ ^[[3]](#footnote-3)^^ and depend on length of the prior treatment. Since we did not control the length of prior treatment, we assumed a relapse rate of 40%. For a power of 0.95, and a two-sample two-tailed t-test, this yielded a N = 71 for the patient group. To account for potential technical errors, we aimed to recruit at least N = 76 patients.

**Supplementary Table 1 – a priori analysis plan**

|  | ***Analysis*** | ***Dependent Variable*** | ***Independent Variable(s)*** | ***Hypothesis*** | ***Statistical Method*** | ***Contrast of Interest*** | ***Intention-to-Treat*** |
| --- | --- | --- | --- | --- | --- | --- | --- |
| *(1)* | *Impulsivity in depressed patients vs healthy controls* | Log *K*  at MA1 | Group: patient vs control | At MA1 *log K* is greater in patients than controls | Two sample *t*-test | Main effect of Group | No |
| *(2)* | *Impulsivity as a function of antidepressant discontinuation* | Log *K* at MA1 and MA2 | Group: early vs late discontinuation | Discontinuation is associated with an increase in *log* *K* between MA1 and MA2 | Linear mixed effects regression modelling | Group x Time interaction | Yes |
| *(3)* | *Impulsivity as a function of questionnaire data* | Log *K*  at MA1 | HAM-D^1^ | HAM-D scores are positively correlated with *log K* | Linear regression modelling, *t*-test on slope | Main effect of HAM-D | No |
|  |  |  | SWLS^1^ | SWLS scores are negatively correlated with *log K* | Linear regression modelling, *t*-test on slope | Main effect of SWLS | No |
|  |  |  | ACE^1^ | ACE scores are positively correlated with *log K* | Linear regression modelling, *t*-test on slope | Main effect of ACE | No |
|  |  |  | HAM-D, ERQ, BSCS, SWLS, ACE, CTQ, TLEQ, MWT-B^1^ | Exploratory analysis: a combination of questionnaire measures (and/or their latent factor structure) is associated with discounting | Linear regression modelling; sparse partial least squares regression, *t*-tests on slope | Main effects of questionnaire measures, or corresponding factors | No |
|  |  |  | HAM-D, ERQ, BSCS, SWLS, ACE, CTQ, TLEQ, MWT-B^1^ | Exploratory analysis: a combination of questionnaire measures (and/or their latent factor structure) *predicts* discounting | Lasso regression modelling; leave one out cross validation within Zurich sample; test prediction on Berlin sample as a hold out | Main effects of questionnaire data, or corresponding factors | No |
| *(4)* | *Relapse rate over time as a function of impulsivity* | Relapse in both patient groups | Log *K* at MA1 | Higher *log K* at MA1 is associated with greater odds of relapse | Cox proportional hazard modelling | *K* x Hazard interaction | Yes |
|  |  |  |  | Higher *log K* at MA1 *predicts* relapse | Lasso regression modelling; leave one out cross validation within Zurich sample; test prediction on Berlin sample as a hold out | Main effect of *K* | No |
| *(5)* | *Relapse over time in the patient group as a function of change in impulsivity following antidepressant discontinuation* | Relapse in early discontinuation patient group | Log *K* at MA1 and MA2 | Increase in *log K* between MA1 and MA2 is associated with greater odds of subsequent relapse | Cox proportional hazard modelling | *K* x Time x Hazard interaction | Yes |
|  |  |  |  | Increase in *log K* between MA1 and MA2 *predicts* subsequent relapse | Lasso regression modelling; leave one out cross validation within Zurich sample; test prediction on Berlin sample as a hold out | *K* x Time interaction | No |
| *(6)* | *Depression score*  *over time as a function of impulsivity* | Hamilton Depression Scale (HAM-D) scores | Log *K* at MA1 | Higher *log K* at MA1 is associated with reduced recovery in HAM-D score over time | Linear mixed effects regression modelling | *K* x Time interaction | Yes |

1. Hamilton Depression Scale (HAM-D), Emotion Regulation Questionnaire (ERQ), Brief Self-Control Scale (BSCS), Daily Hassles, Satisfaction with Life Scale (SWLS), Adverse Childhood Experience (ACE), Childhood Trauma Questionnaire (CTQ), Traumatic Life Events Questionnaire (TLEQ), Mehrfachwahl-Wortschatz-Intelligenztest (MWT-B).

**Supplementary Table 2 – *log K* differences with respect to potential confounders**

| Potential confounder | t statistic/Spearman $\rho$/F statistic | Degrees of freedom | P value | Cohen’s d effect size |
| --- | --- | --- | --- | --- |
| Gender (Male vs. Female) | t=0.24 | 149 | 0.806 | 0.04 |
| Site (Zurich vs. Berlin) | t=1.72 | 149 | 0.086 | 0.30 |
| Treated by general physician (GP) vs. not treated by GP | t=0.49 | 149 | 0.625 | 0.09 |
| Treated by psychiatrist vs. not treated by psychiatrist | t=0.31 | 149 | 0.754 | 0.05 |
| Treated by psychologist vs. not treated by psychologist | t=0.65 | 149 | 0.511 | 0.14 |
| Medication classes (ANOVA) | F=0.84 | 2,94 | 0.431 | - |
| Chronicity | Spearman’s $\rho=$0.07 | - | 0.330 | - |

*Note*. We tested for associations between *log K* at baseline (MA1) and different potential confounding factors, namely: gender group, site (Zurich/Berlin), treatment group (treated by general physician, psychiatrist, or psychologist), medication class and chronicity of depression, where the latter is defined as the number of months sick within the last five years Associations with dichotomous variables were tested using two-sample *t*-tests, categorical variables by one-way ANOVA. We also tested a correlation between baseline *log K* and chronicity of depression, a continuous variable. This step was not explicitly stated in the original analysis plan.

**Supplementary Table 3 – results of linear regression model having *log K* at baseline as dependent variable and depression rating questionnaire scores as independent variables**

| Questionnaire | Coefficient estimate | 95% confidence interval | t-statistic | Degrees of freedom | P value |
| --- | --- | --- | --- | --- | --- |
| ACE | -0.032 | [-0.286,0.221] | -0.25 | 142 | 0.802 |
| SWLS | 0.001 | [-0.061,0.064] | 0.04 | 142 | 0.961 |
| HAM-D | 0.198 | [0.042,0.354] | 2.51 | 142 | 0.013 |
| ERQ | 0.067 | [-0.107,0.241] | 0.75 | 142 | 0.448 |
| BSCS | 0.016 | [-0.023,0.055] | 0.81 | 142 | 0.414 |
| CTQ | 0.019 | [-0.018,0.057] | 1.03 | 142 | 0.304 |
| TLEQ | -0.011 | [-0.061,0.037] | -0.47 | 142 | 0.637 |
| MWT-B | -0.060 | [-0.123,0.001] | -1.92 | 142 | 0.057 |

**Supplementary Table 4 – sparse partial least squares with *log K* at baseline as dependent variable and depression rating questionnaire scores as independent variables**

| Questionnaire | Coefficient estimate | VIP score |
| --- | --- | --- |
| ACE | -0.032 | 0.209 |
| SWLS | 0.016 | 0.375 |
| HAM-D | 0.198 | 1.683 |
| ERQ | 0.067 | 0.518 |
| BSCS | 0.016 | 0.591 |
| CTQ | 0.019 | 1.249 |
| TLEQ | -0.011 | 0.677 |
| MWT-B | -0.060 | 1.530 |

*Note*. In line with our a priori analysis plan, we also fitted a partial least-squares model, using the MATLAB plsregress function. The latter approach finds linear combinations of the independent variables (depression questionnaires), that explain variance in the dependent variable (log K) and allows to further examine the importance of each explanatory predictor using the variable importance in projection (VIP) measure^[[4]](#footnote-4)^.

Fitting a partial least squares regression to the data, where the logarithm of the discount rate is the dependent variable, and the scores to the depression rating questionnaire are the predictor variables explained 9.01% of the variance in the logarithm of discount rate. HAM-D, CTQ, and MWTB scores had the largest VIP scores among all 8 rating scales (VIP=1.683,1.249,1.530 respectively).

**Supplementary Table 5 – means and standard deviations of group comparisons**

| **Variable** | **Mean (Median)** | **Standard deviation (Interquartile range)** |
| --- | --- | --- |
| Log K Baseline Non-relapse | -5.167 | 1.522 |
| Log K Baseline Relapse | -5.269 | 1.697 |
| [Log K MA2-Log K MA1] Non-relapse | 0.215 | 1.139 |
| [Log K MA2-Log K MA1] Relapse | 0.039 | 1.263 |
| [Log K MA2-Log K MA1] MA1MA2D | 0.157 | 1.036 |
| [Log K MA2-Log K MA1] MA1DMA2 | 0.162 | 1.154 |
| [HAM-D MA1] MA1MA2D | 1.979 (2) | 1.824 (1) |
| [HAM-D MA2] MA1MA2D | 2.178 (2) | 1.600 (2) |
| [HAM-D MA1] MA1DMA2 | 1.652 (1) | 1.876 (3) |
| [HAM-D MA2] MA1DMA2 | 3.163 (3) | 2.645 (4) |
| Log K Baseline Controls | -5.597 | 1.627 |
| Log K Baseline Patients | -5.042 | 1.605 |
| HAM-D MA1 Controls | 0.377 (0) | 0.814 (0.25) |
| HAM-D MA1 Patients | 1.813 (1) | 1.932 (3) |

*Note.* Medians and interquartile ranges are reported in brackets for groups where a Bartlett test rejects the null hypothesis that both groups come from normal distributions with equal variances.

**Figure S1 Distribution of *log K* in relapsers and non-relapsers**


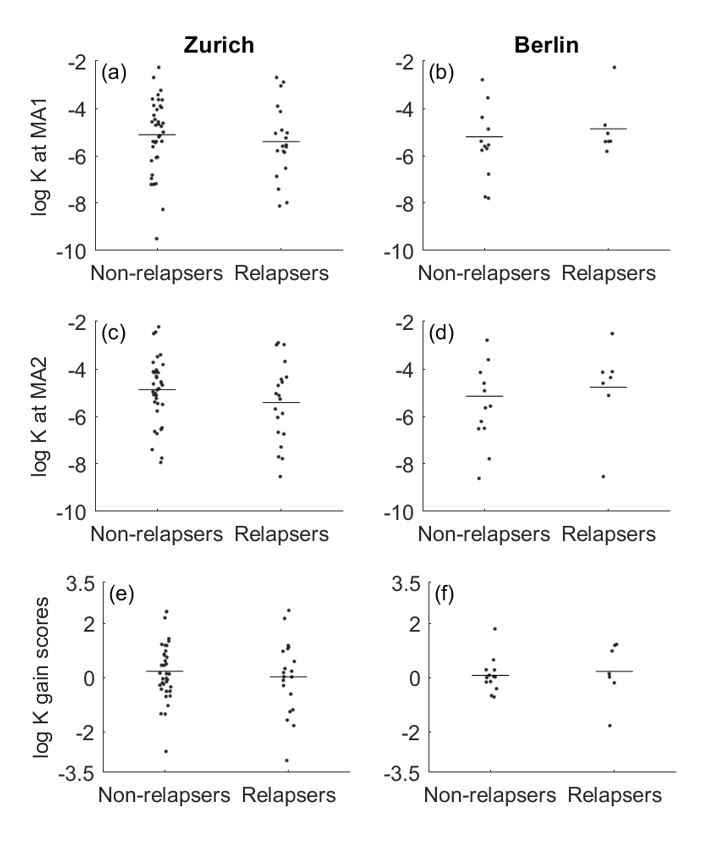


Note. Scatter plots of individual log K values in remitted patients who relapsed during follow up (“Relapsers”) and patients who did not (“Non-relapsers”), in Zurich (a,c,e) and Berlin (b,d,f) sites, at either MA1 (a,b), MA2 (c,d) and gain scores (e,f). Horizontal lines mark the average log K value of the corresponding group.

**Figure S2 Correlations between *log K* at baseline and rating subscales**


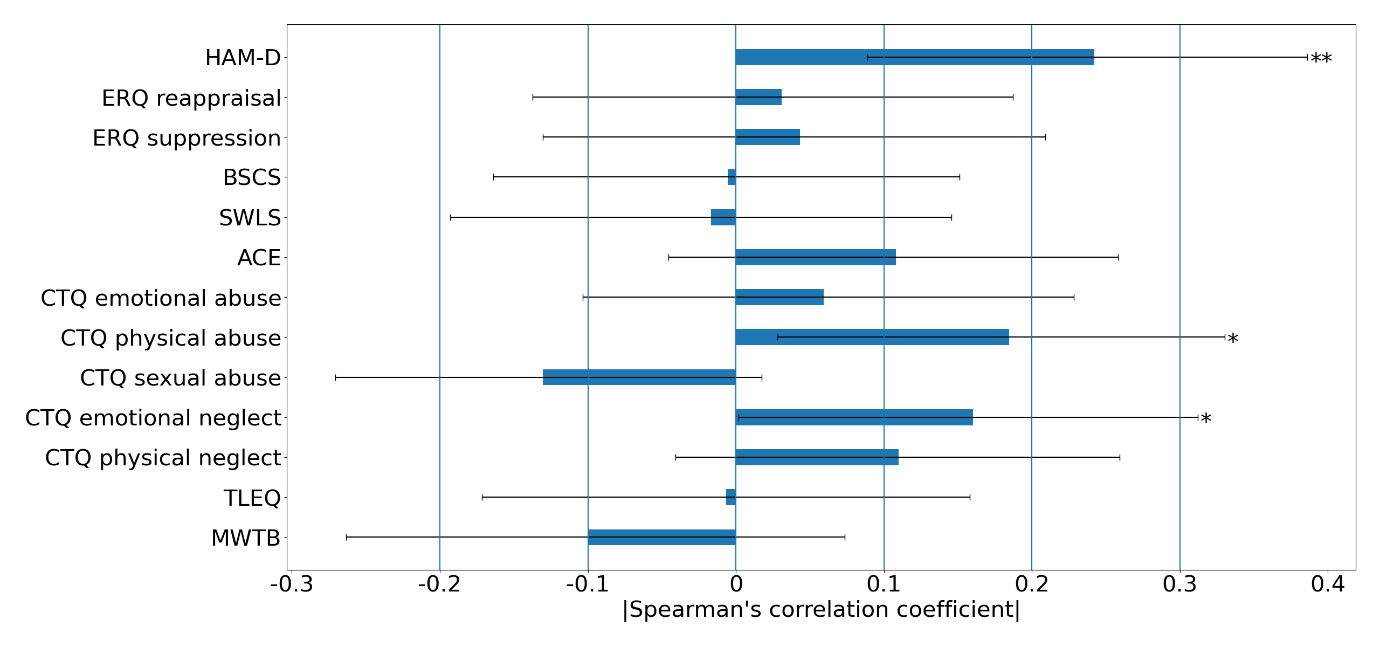


Note. HAM-D=Hamilton Depression Scale, ERQ=Emotion Regulation Questionnaire (ERQ), BSCS=Brief Self-Control Scale, SWLS=Satisfaction with Life Scale, ACE=Adverse Childhood Experience, CTQ=Childhood Trauma Questionnaire, TLEQ=Traumatic Life Events Questionnaire, MWTB=Mehrfachwahl-Wortschatz-Intelligenztest. Error bars represent 95% confidence interval for Spearman’s correlation coefficient estimated using 10,000 bootstrap iterations. Group difference p-value: * .01<p<.05, ** .001<p<.01.

1. Berwian, I. M., Wenzel, J. G., Collins, A. G., Seifritz, E., Stephan, K. E., Walter, H., & Huys, Q. J. (2020). Computational mechanisms of effort and reward decisions in patients with depression and their association with relapse after antidepressant discontinuation. *JAMA psychiatry*, *77*(5), 513-522.‏ [↑](#footnote-ref-1)
2. Geddes, J. R., Carney, S. M., Davies, C., Furukawa, T. A., Kupfer, D. J., Frank, E., & Goodwin, G. M. (2003). Relapse prevention with antidepressant drug treatment in depressive disorders: a systematic review. *The Lancet*, *361*(9358), 653-661.‏ [↑](#footnote-ref-2)
3. Hollon, S. D., Shelton, R. C., Wisniewski, S., Warden, D., Biggs, M. M., Friedman, E. S., ... & Rush, A. J. (2006). Presenting characteristics of depressed outpatients as a function of recurrence: preliminary findings from the STAR* D clinical trial. *Journal of psychiatric research*, *40*(1), 59-69.‏ [↑](#footnote-ref-3)
4. S Wold, A Johansson, M Cochi (eds). *PLS-partial least squares projections to latent structures*. ESCOM Science Publishers: Leiden, 1993; 523–550. [↑](#footnote-ref-4)
